# Feasibility of Crosslinked Acrylic Shape Memory Polymer for a Thrombectomy Device

**DOI:** 10.1155/2014/971087

**Published:** 2014-02-25

**Authors:** Andrea D. Muschenborn, Keith Hearon, Brent L. Volk, Jordan W. Conway, Duncan J. Maitland

**Affiliations:** Biomedical Engineering Department, Texas A&M University, 3120 TAMU, College Station, TX 77843, USA

## Abstract

**Purpose:**

To evaluate the feasibility of utilizing a system of SMP acrylates for a thrombectomy device by determining an optimal crosslink density that provides both adequate recovery stress for blood clot removal and sufficient strain capacity to enable catheter delivery.

**Methods:**

Four thermoset acrylic copolymers containing benzylmethacrylate (BzMA) and bisphenol A ethoxylate diacrylate (Mn~512, BPA) were designed with differing thermomechanical properties. Finite element analysis (FEA) was performed to ensure that the materials were able to undergo the strains imposed by crimping, and fabricated devices were subjected to force-monitored crimping, constrained recovery, and bench-top thrombectomy.

**Results:**

Devices with 25 and 35 mole% BPA exhibited the highest recovery stress and the highest brittle response as they broke upon constrained recovery. On the contrary, the 15 mole % BPA devices endured all testing and their recovery stress (5 kPa) enabled successful bench-top thrombectomy in 2/3 times, compared to 0/3 for the devices with the lowest BPA content.

**Conclusion:**

While the 15 mole% BPA devices provided the best trade-off between device integrity and performance, other SMP systems that offer recovery stresses above 5 kPa without increasing brittleness to the point of causing device failure would be more suitable for this application.

## 1. Introduction

Thrombectomy devices are utilized endovascularly to remove blood clots and reestablish blood flow in an occluded artery. When the occluded artery is part of the neurovasculature, failure to reestablish blood flow in a timely manner results in a significant reduction in the oxygenation of brain tissue. Such an event is called acute ischemic stroke and occurs approximately every minute to someone in the US [1–3]. Of all ischemic stroke cases, 8–12% are fatal, and the 6- month poststroke period for survivors renders this disease the leading cause of adult long-term disability [1, 2, 4, 5]. To date, there is one drug, tissue plasminogen activator (tPA), and four devices, Merci Retriever (Stryker, California), Penumbra System (Penumbra Inc., California), Solitaire FR (ev3, California), and Trevo Retriever (Stryker, California), that have been approved by the US Food and Drug Administration (FDA) for the treatment of acute ischemic stroke [2, 3, 6]. However, the success rates of these clinical treatments remain significantly limited. Only 2–3% of ischemic stroke patients are eligible to receive tPA because of its narrow treatment time window and other exclusion criteria [6, 7]. Consequently, tPA-ineligible patients are usually treated with one of the approved thrombectomy devices, which are often associated with other complications, including blood clot dislodgement and distal migration, limited device maneuverability, and arterial perforation [2, 8–10]. There remains a need to evaluate alternative materials and device designs that can enable rapid and successful reestablishment of blood flow in ischemic stroke patients.

The emergence of smart materials has given rise to the investigative development of numerous minimally invasive endovascular devices that exploit novel material capabilities. One such capability is shape memory, which is a material’s ability to store temporary shape(s) and then actuate to a primary geometry when exposed to stimuli such as heat or moisture [11–13]. Shape memory alloys (SMAs), particularly nickel titanium (nitinol), have been used in the biomedical field for over 20 years [14]. Three out of the four FDA-approved devices for the treatment of ischemic stroke are made with nitinol. For example, the Merci Retriever is a nitinol wire that has been preshaped with a corkscrew geometry. The other two nitinol-based thrombectomy devices are self-expanding stents, Solitaire FR and Trevo. However, one major drawback of these devices is their length, which limits their placement and maneuverability during treatment [15].

Shape memory polymers (SMPs) are being investigated as alternative candidate materials for endovascular applications such as thrombectomy, primarily because SMPs exhibit recoverable deformations up to 100 times greater than those reported for SMAs [13, 16, 17]. Consequently, many SMP-based endovascular devices are capable of undergoing the significant shape changes necessary for catheter delivery [11, 18–22]. This property enables the exploration of novel device designs that have the potential to address the issues with commercially available thrombectomy devices, for example, difficult maneuverability. For example, Buckley et al. [23] proposed a design that is crimped to a cylinder for catheter delivery and is actuated to a flower shape distal to the occlusion such that only the edge of the “petals” is in contact with the vessel during treatment minimizing device-vessel contact points.

While SMPs generally exhibit maximum recoverable forces less than 1/100 times the recovery force of SMAs, another advantage is tunability of thermomechanical properties including recovery force, modulus, actuation temperature (for thermally actuated SMPs), and recoverable strain to meet the material demands of various applications. One effective, demonstrated method of tailoring thermomechanical properties of SMPs is controlling crosslink density [24, 25]. Increasing crosslink density of SMP systems has been generally shown to increase recovery stress [11, 26, 27]. At the same time, however, increasing crosslink density can also have undesired effects such as increased brittleness and decreased strain capacity [28].

The objective of this study is to evaluate the feasibility of fabricating SMP-based thrombectomy devices from crosslinked SMP acrylates by (1) finding the crosslink density that results in increased recovery stress without compromising the strain capacity necessary for crimping and actuation and (2) determining whether the resulting thermomechanical properties would be suitable for thrombectomy in a bench-top model.

## 2. Materials and Methods

After finalizing the device design, the deformation of the device was modeled via finite element analysis (FEA) to estimate maximum strains. Then, a system of crosslinked acrylic SMPs was designed with varying crosslink densities and with strain capacities greater than the maximum strains predicted by FEA. Devices were subsequently machined and subjected to force-monitored crimping and constrained recovery, and finally blood clot removal experiments were performed in a bench-top thrombotic model.

### 2.1. Device Design

**Figure 1:**
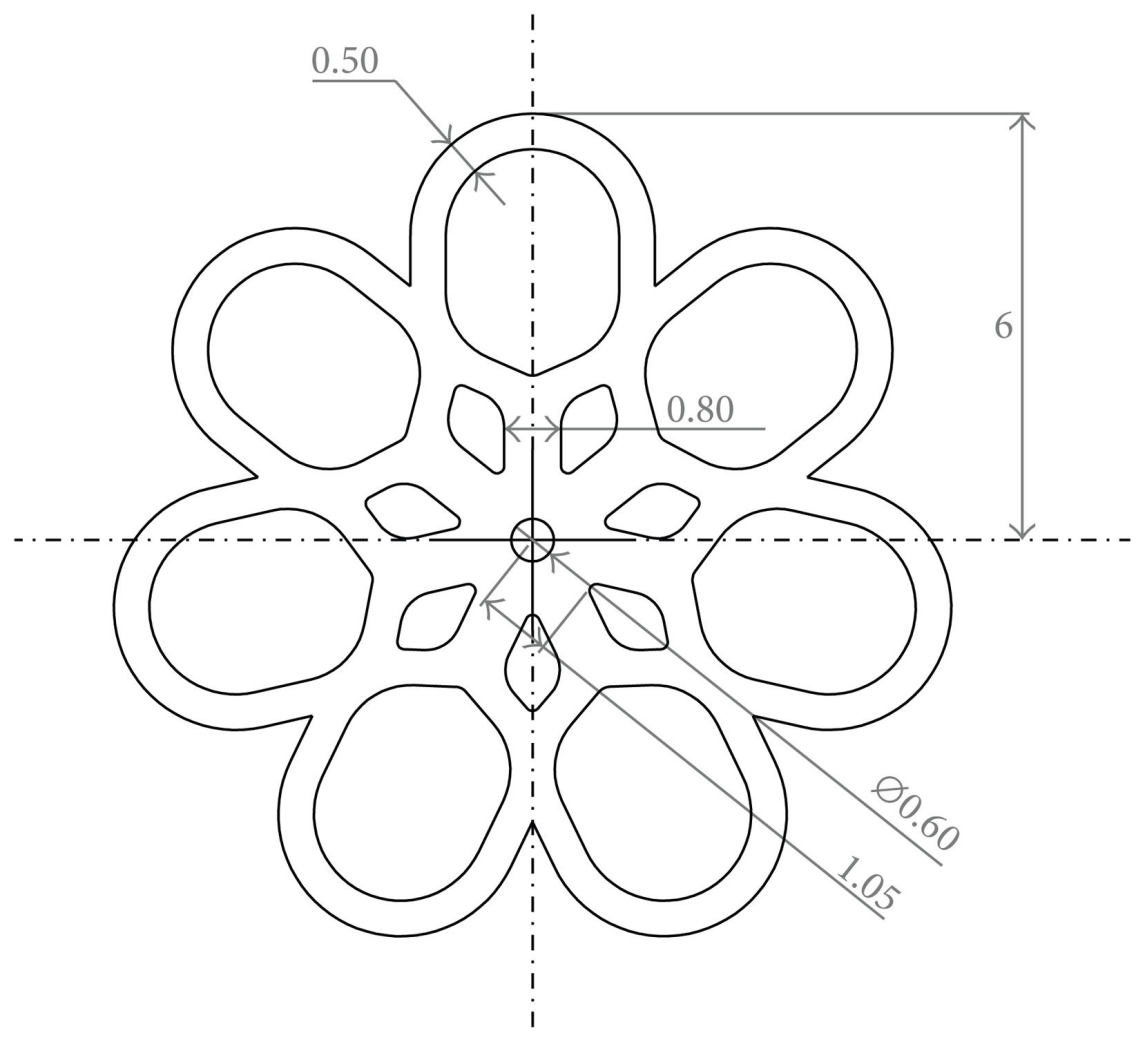
Flower-shaped thrombectomy device designed in Solid-Works with dimensions in mm. This design was adapted from an original thrombectomy device proposed by Buckley et al. [23].

Flower-shaped devices were designed in SolidWorks based on the geometry proposed by Buckley et al. [23]. Modifications to the design were necessary to prevent portions of the device folding over themselves during crimping and to reduce potential areas of stress concentrations. In order to better visualize the devices during testing, we designed them to be 4 times larger than they would be for *in vivo* testing. The design used in this study is depicted in Figure 1.

### 2.2. Finite Element Analysis Simulations

The device design was imported from the SolidWorks file created to fabricate the device (see Figure 1), and the crimping funnel (small inner diameter = 3.5 mm, large inner diameter = 12.5 mm) and ball (outer diameter = 2 mm) were created in Abaqus as analytical rigid parts. The device was modeled using 1/14th symmetry in a cylindrical coordinate system. The device was meshed using approximately 12000 linear, 3D, hybrid brick elements (C3D8H) and was modeled as an isotropic Mooney-Rivlin material with coefficients of C_10_ = 3.06865 MPa and C_01_ = −0.94798 MPa. These coefficients were obtained by analyzing, using the material evaluator function in Abaqus, the tensile test data for each of the polymer compositions and averaging the resulting Mooney-Rivlin coefficients. Because the strain—which is dictated by the geometry change in passing through the funnel—of the thrombectomy device was the primary quantity of interest, efforts were not made to further optimize the constitutive response to the tensile test data. The nonlinear geometry feature was enabled for the simulation, and “hard” contact (i.e., no penetration) was assumed between the funnel and the device, the ball and the device, and the analytical planes and the device (to model self-contact in the loops during the crimping). A coefficient of friction of 0.04 was assigned between the funnel and the device based on a bench-top friction test between blocks of polytetrafluoroethylene (PTFE) and poly(methyl methacrylate) (PMMA). A value of 0.8 was assigned as the coefficient of friction between the device and the analytical planes (for self-contact) and between the ball and the device (to account for roughness of the ball) based on published data of the nominal coefficient of friction in acrylic [29]. The ball, device, and funnel were initially concentrically positioned, and then the ball was held spatially fixed while the funnel was moved to encompass the device.

### 2.3. Polymer Synthesis

The monomer benzyl methacrylate (BzMA), the crosslinker bisphenol A ethoxylate diacrylate (Mn~512, BPA), and the photoinitiator 2,2-dimethoxy-2- phenylacetophenone (DMPA) were purchased from Sigma Aldrich and used as received without further purification. This monomer and difunctional crosslinker combination, which is similar to that reported by Safranski and Gall [24], was selected because it enables the synthesis of a series of SMPs with tailorable crosslink densities and glass transitions above body temperature in the range of 65 to 75°C. Benzyl methacrylate was also selected because Safranski and Gall [24] demonstrated that poly(benzyl methacrylate) thermoset SMPs have higher toughness than numerous other thermoset acrylics. Four thermoset acrylic BzMA-BPA copolymers containing 5, 15, 25, and 35 mole% BPA and 0.5 weight% DMPA were prepared in 51 × 76 × 0.4 mm sheets by bulk UV curing. After massing, the monomer, crosslinker, and photoinitiator mixtures were injected between 51 × 76 mm glass slides coated with Rain-X separated by 0.4 mm spacers and cured for 99 minutes using 365nm UV irradiation inside a UVP CL-1000 crosslinking chamber. The cured thermoset films were then postcured at 130°C with vacuum at 1 torr for 12 hours and subsequently stored under desiccation.

### 2.4. Dynamic Mechanical Analysis

Dynamic mechanical analysis (DMA) experiments were carried out in tension using a TA Instruments Q800 dynamic mechanical analyzer and 4 × 30 × 0.4 mm rectangular specimens (*n* = 5). The rectangular specimens were machined with a 40W Gravograph LS100 CO_2_ laser machining device using a 38.1 mm lens, a speed setting of 5, a power setting of 10, and a laser resolution of 1200 dots per inch. Specimens were cleaned with a methanol damp Kimwipe, driedat 90° C under vacuum at 1 Torr, and subsequently stored under desiccation prior to being tested. DMA experiments were carried out from −20 to 140°C in the DMA multifrequency/strain instrument mode using a frequency of 1Hz, a strain of 0.1%, a preload force of 0.01N, a force track of 150%, and a heating rate of 2°C/min. Data were recorded using TA Instruments Q-series software and analyzed using TA Instruments Universal Analysis software.

### 2.5. Tensile Testing

Uniaxial tensile testing experiments were conducted to failure on ASTM type V dog bone samples (*n* = 5) using a dual-column Instron model 5965 tensile tester with a 500N load cell, 1000N high temperature pneumatic grips, and a temperature chamber thermally controlled by forced convection heating. The dog bone samples were cut using a 40W Gravograph LS100 CO_2_ laser machining device with the same instrument parameters used to cut the rectangular DMA specimens. Specimens were cleaned with a methanol damp Kimwipe, dried at 90°C under vacuum at 1 Torr, and subsequently stored under desiccation prior to being tested. Specimen deformation was measured optically using an Instron Advanced Video Extensometer with a 60 mm field-of-view lens. Specimens were heated to target temperatures under zero loads (unclamped bottom grip) and were held isothermally for 30 minutes to allow thermal equilibrium to be reached. The bottom grip was then clamped, and the strain-to-failure experiments were subsequently begun using a deformation rate of 10 mm/min. Data were recorded using Instron Bluehill 3 software.

### 2.6. Device Fabrication

The postcured 51 × 76 × 0.4 mm sheets of the four different copolymers were cut with the design pattern shown in Figure 1 utilizing the 40W Gravograph LS100 CO_2_ laser machining device. The same instrument parameters were implemented as the ones used to cut the DMA and dog bone specimens. Devices were carefully cleaned with a methanol damp Kimwipe, dried at 90°C under vacuum at 1 Torr prior, and subsequently stored under desiccation prior to being tested.

### 2.7. Device Crimping

**Figure 2:**
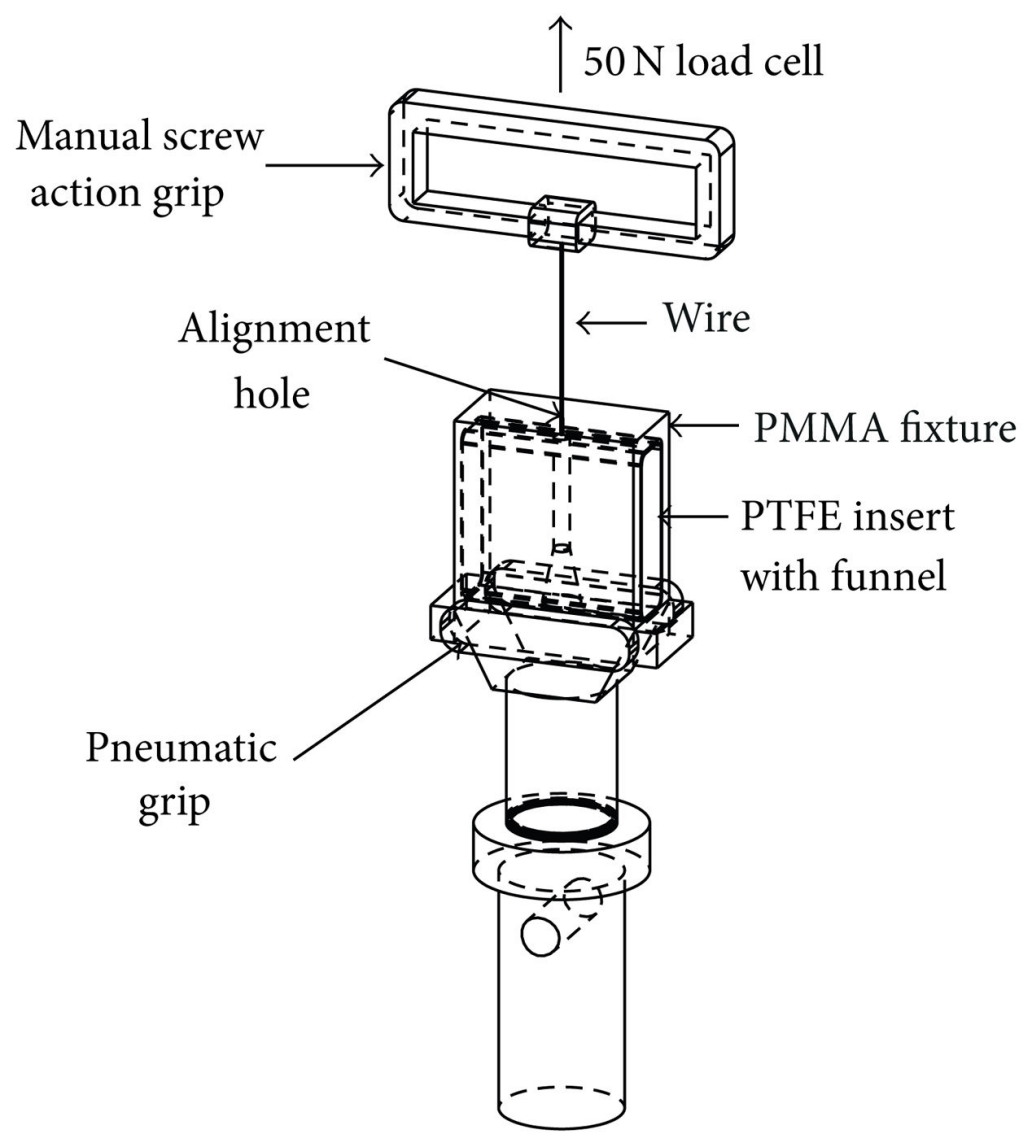
Sketch of custom-made apparatus for the crimping of the thrombectomy devices. The apparatus was designed so that it was compatible with the Instron tensile tester. Each device was inserted in the PTFE funnel, and after thermal equilibrium was achieved after 1 hour, the device was slid upwards by the movement of the crosshead connected to the manual screw action grip. The device was allowed to cool down in the narrow cylindrical feature of the PTFE insert, above the funnel.

A custom fixture compatible with Instron tensile tester systems was fabricated with a minicomputer numerically controlled (CNC) milling machine (see Figure 2). It consisted of a removable portion made of PTFE with a funnel-like geometry on the inside and a stationary portion made of PMMA that was attached to the tensile tester’s bottom pneumatic grip. The maximum diameter of the funnel was 12.5 mm and it contracted gradually to 3.5 mm (the portion with the smallest diameter was 1 cm long and it served to fit the length of the crimped device and allow it to cool down). The stationary portion had a cap with a 0.3 mm hole machined with an excimer laser that helped center the wire that held the devices throughout the crimping process. This wire had ~2mm diameter ball of 96.5 Sn 3.5 Ag solder purchased from Indium Corporation at the tip to hold the devices as they were pulled during crimping. By inserting a thermocouple inside the funnel of the assembled crimping fixture, it was determined that in order to achieve the target temperature inside the funnel within 45 minutes the temperature chamber was required to be set 4°C higher. Thus, devices were allowed to thermally equilibrate inside the funnel for a minimum of 1 hour prior to testing. The tensile tester had a 50N load cell, rated with a resolution of 0.00025N, and was set to extension mode at a strain rate of 5mm/min. Data were recorded using Instron Bluehill 3 software. The crimping experiments were performed at temperatures corresponding to the mechanical transition temperatures, DMA loss modulus peak, and tan delta peak, of each copolymer’s composition (*n* = 5).

### 2.8. Device Constrained Recovery

**Figure 3:**
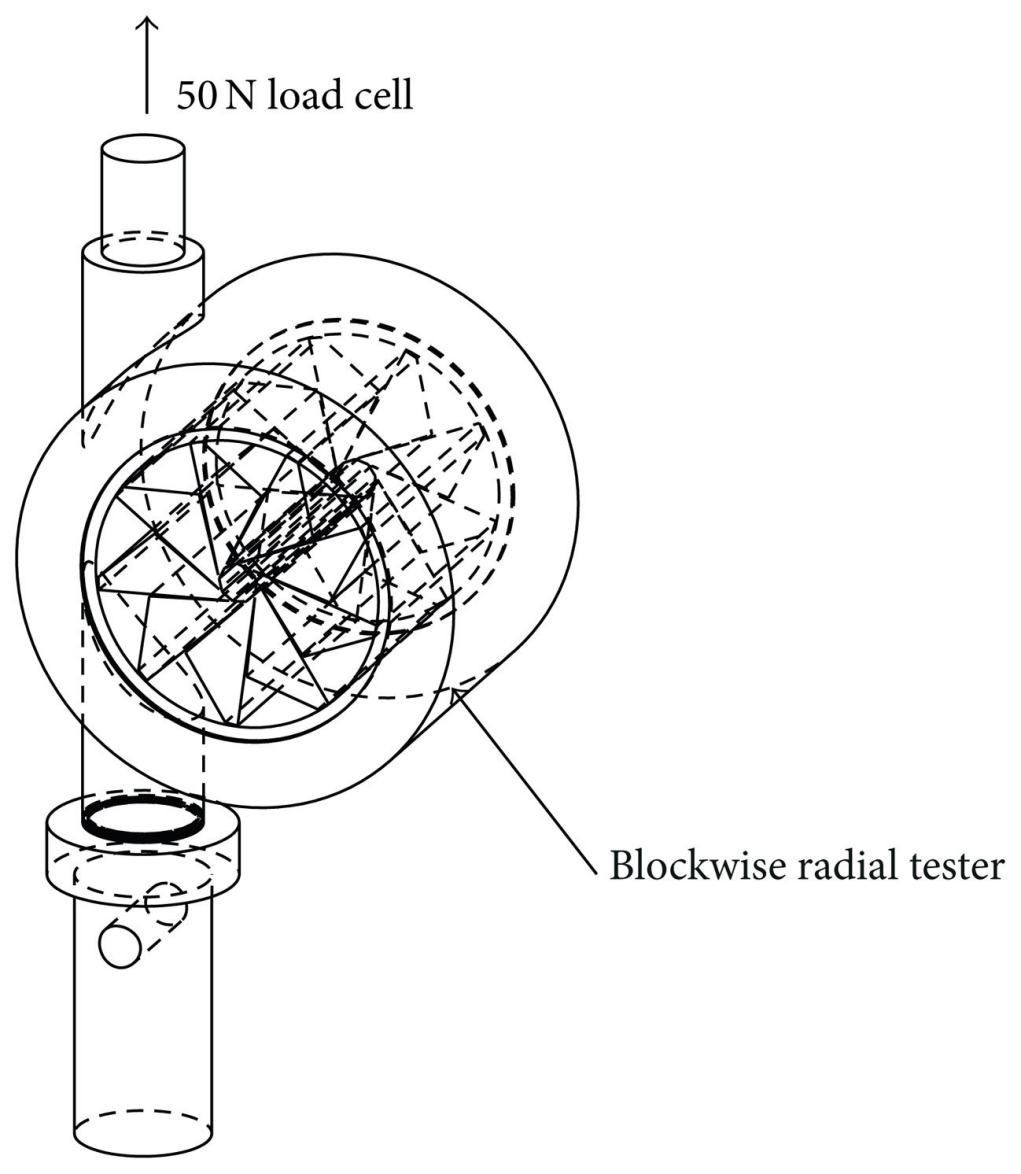
Sketch of Blockwise radial tester utilized in the constrained recovery experiments (not drawn to scale). The diameter enclosed by the blades was set with a high accuracy gage pin, and each device was inserted in its crimped configuration. The temperature of the blades was ramped from 30 to 55°C, while the diameter of the radial tester was kept constant. As the thrombectomy device recovered its shape, it exerted a load onto the blades, which was sensed by the load cell.

A commercially available Blockwise Engineering RJA62 radial stress tester compatible with Instron tensile tester systems with a 50N load cell was utilized to perform constrained recovery measurements of the crimped devices (see Figure 3). The radial tester temperature was ramped from 30°C to 55°C and the recovery stress of the devices that were previously crimped at loss modulus peak temperature was measured at 1Hz using Instron Bluehill 3 software. A thermocouple was installed in close proximity of each device, and temperature was also recorded at 1Hz using LabView. The diameter of the tester was kept constant at 3.75 mm after setting it with a high accuracy gage pin. The blade heating effects of the radial tester were accounted for by subtracting data measured with the empty tester from the data of all measured devices for the same temperature range.

### 2.9. Bench-Top Blood Clot Removal

**Figure 4:**
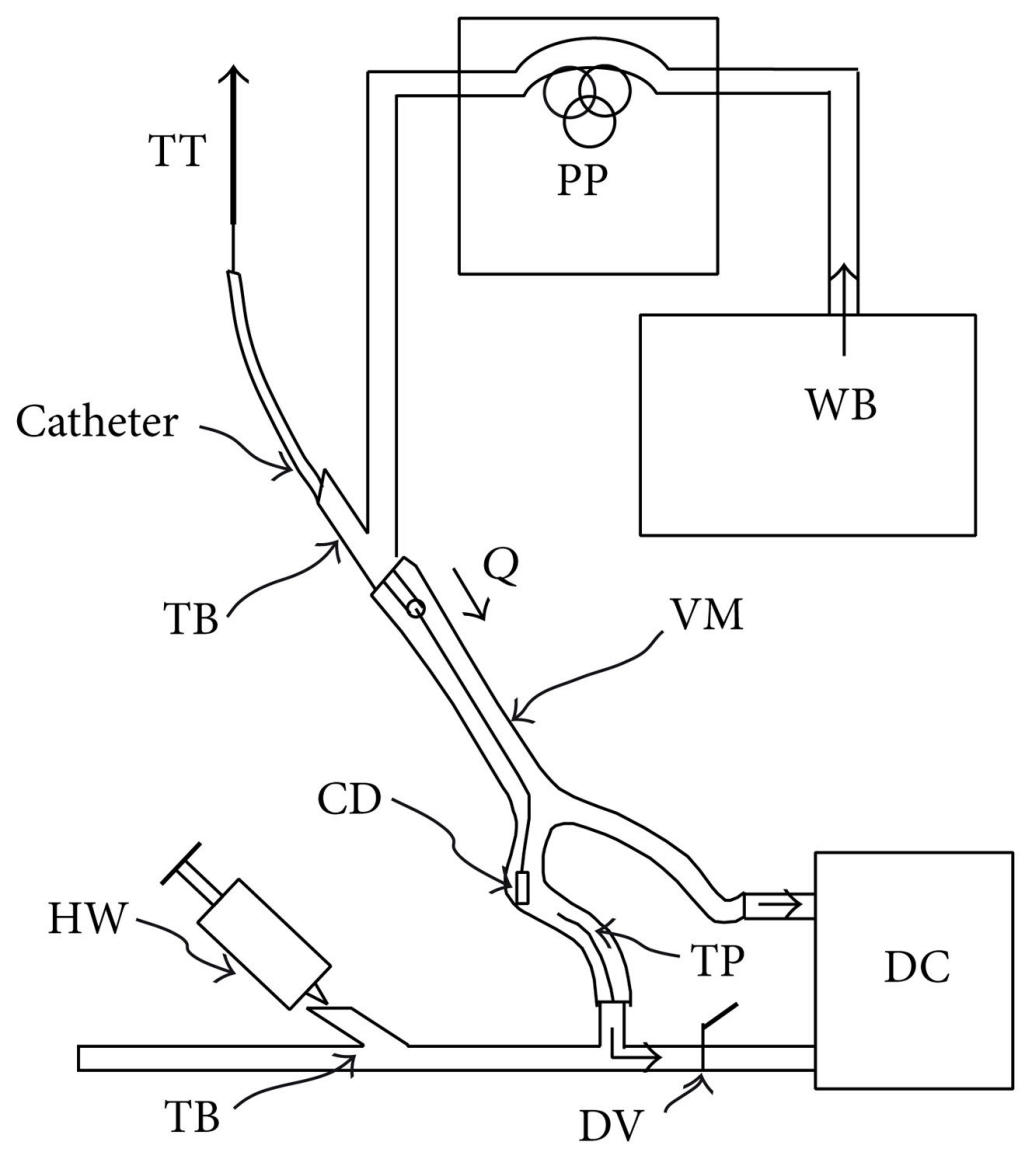
Bench-top thrombotic stroke model. WB: water bath (37°C); PP: peristaltic pump; TT: tensile tester (extension rate = 75 mm/min); *Q*: experimental flow rate (=57 mL/min); TB: Touhy Borst valve; CD: crimped device location; VM: vessel model; TP: thermocouple probe; HW: hot water syringe; DV: discharge valve; DC: discharge container.

A bench-top thrombotic stroke model shown in Figure 4 was constructed to test the feasibility of the SMP flower-shaped devices for blood clot hold during removal. The blood clots were prepared with bovine blood (10 mL), which was acquired from Vet Med Park, College Station, Texas. Barium sulphate (1 g) and bovine thrombin (25 IU), both purchased from VWR International, were added to the bovine blood, and the mixture was injected into a silicone tube with 10 mm inner diameter. The mixture was gently agitated for about 5 minutes and was incubated at room temperature for at least 1 hour prior to testing [30]. Blood clots of roughly 2 cm in length were cut with a scalpel.

Each crimped device was inserted into the silicone bifurcated vessel model (purchased from Shelley Medical Imaging Technologies, Ontario, Canada) followed by a blood clot. Water at 37°C was pumped into the main vessel segment (lumen diameter = 8 mm) at a flow rate of 57 mL/min via a peristaltic pump. The *in vivo* dynamically similar flow rate via the matching of the Reynolds number that corresponds to the experimental flow rate is within the range of flow rates recorded in the middle cerebral artery, a common site for thromboembolic occlusion in the neurovasculature [31]. Each device was actuated by injecting water near boiling temperature with a syringe while shutting the valve that connects one of the bifurcations of the vessel model to the discharge container. The device was attached to a guide wire, which was connected to a Synergy MTS tensile tester through a catheter. To ensure the repeatability of device testing, the crosshead of the tensile tester pulled each device at an extension rate of 75 mm/min to remove the blood clot. The temperature before and during actuation was recorded at 1Hz by placing a thermocouple near the location of the device.

## 3. Results

### 3.1. FEA Simulations

**Figure 5:**
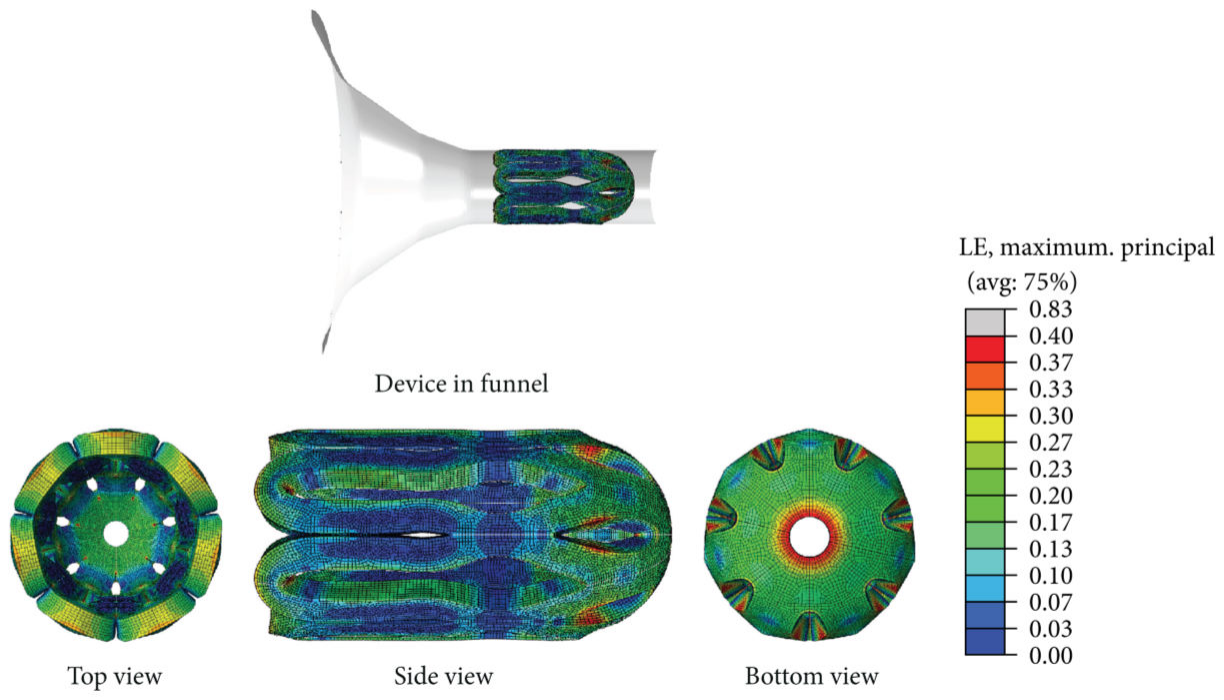
Estimates in the thrombectomy device for the principal logarithmic strains during the crimping procedure. The maximum principal strains are approximately 40% and are located on the struts of the bottom loops of the device as well as near the center circle.

Finite element simulations were performed using Abaqus 6.12-1 finite element software to estimate the strains imposedon the thrombectomy device during the crimping procedure. The geometry of the thrombectomy device, adapted from Buckley et al. [23], was imported from SolidWorks, and the additional parts necessary for the crimping—specifically, a funnel with a final inner diameter of 3.5 mm and a rigid sphere with a diameter of 2 mm— were created as analytical rigid parts in Abaqus. The ball was placed in contact with the thrombectomy device and the funnel was moved so as to crimp the device around the rigid ball. This motion mimicked that of pulling the device through the funnel using a wire that was terminated by a 2 mm ball. Figure 5 shows the strain contours on the device when it was in the final crimped state inside the funnel. In this figure, the device, which was modeled using 1/14th symmetry about the longitudinal axis, was mirrored and then patterned 7 times to be displayed in its entirety. The maximum principal strains were approximately 0.35–0.4 mm/mm and were located at the base of the device near the circular cutout as well as on the struts of the innermost loops. The strain concentrations near the center hole of the device were a result of the large deformation necessary to bend the petals upward, and the concentrations on the struts were a result of the twisting necessary to align portions of the petals for insertion into the funnel.

### 3.2. Material Properties of Copolymers

**Figure 6:**
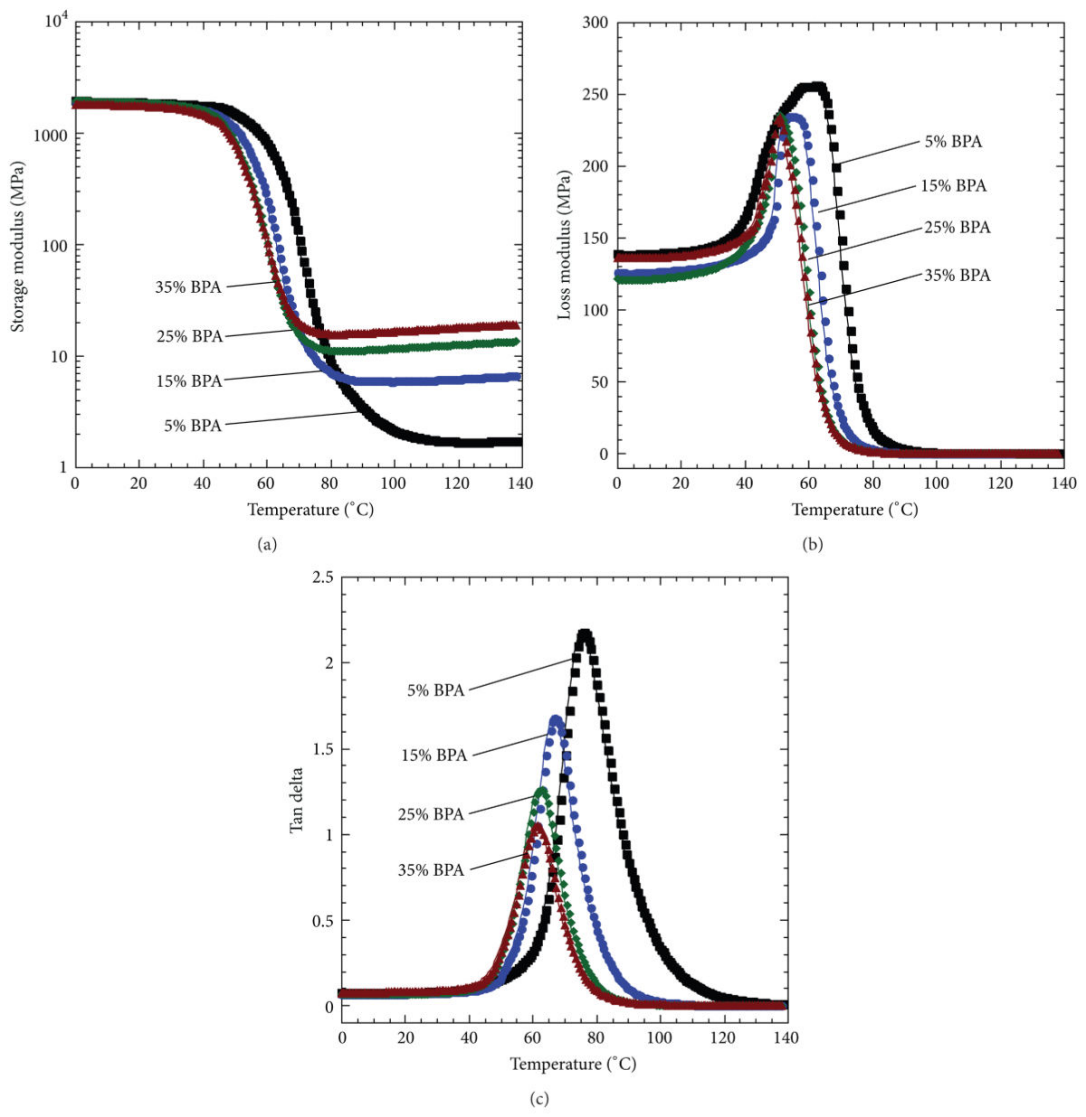
Dynamic mechanical response of the four copolymers, storage modulus (a), loss modulus (b), and tan delta (c). In panel (a), the lowest point of the curve (i.e., the rubbery modulus) increased as a function of increasing crosslinker. Additionally, an increase in the temperature that corresponds to loss modulus peak and tan delta peak in panels (b) and (c), respectively, was observed as a result of increasing crosslinker.

Plots of storage modulus, loss modulus, and tan delta for acrylic SMP samples containing 5, 15, 25, and 35 mole% BPA are provided in Figures 6(a)–6(c), and numerical values for rubbery modulus, loss modulus peak temperatures, and tan delta peak temperatures are provided in Table 1. Rubbery moduli were defined as the lowest value in the rubbery storage modulus curves for all four chemical compositions. As BPA crosslinker was increased from 5 to 35 mole%, rubbery modulus increased from 1.6 to 15.6 MPa. Loss modulus peak temperatures were in the range of 50.8 to 62.3°C, tan delta peak temperatures were in the range of 61.5 to 72.3°C, and both loss modulus peak temperatures and tan delta peak temperatures decreased with increasing BPA crosslinker composition. Although increasing crosslink density often results in increased glass transition temperature in thermoset polymers, the decrease in glass transition with increasing BPA composition in this study was expected because the BPA crosslinker contains flexible polyethylene oxide repeat units, which have a competing effect with crosslink density on glass transition. It was this competing effect that was used to enable the synthesis of four SMPs with significantly different crosslink densities and similar glass transition temperatures.

**Figure 7:**
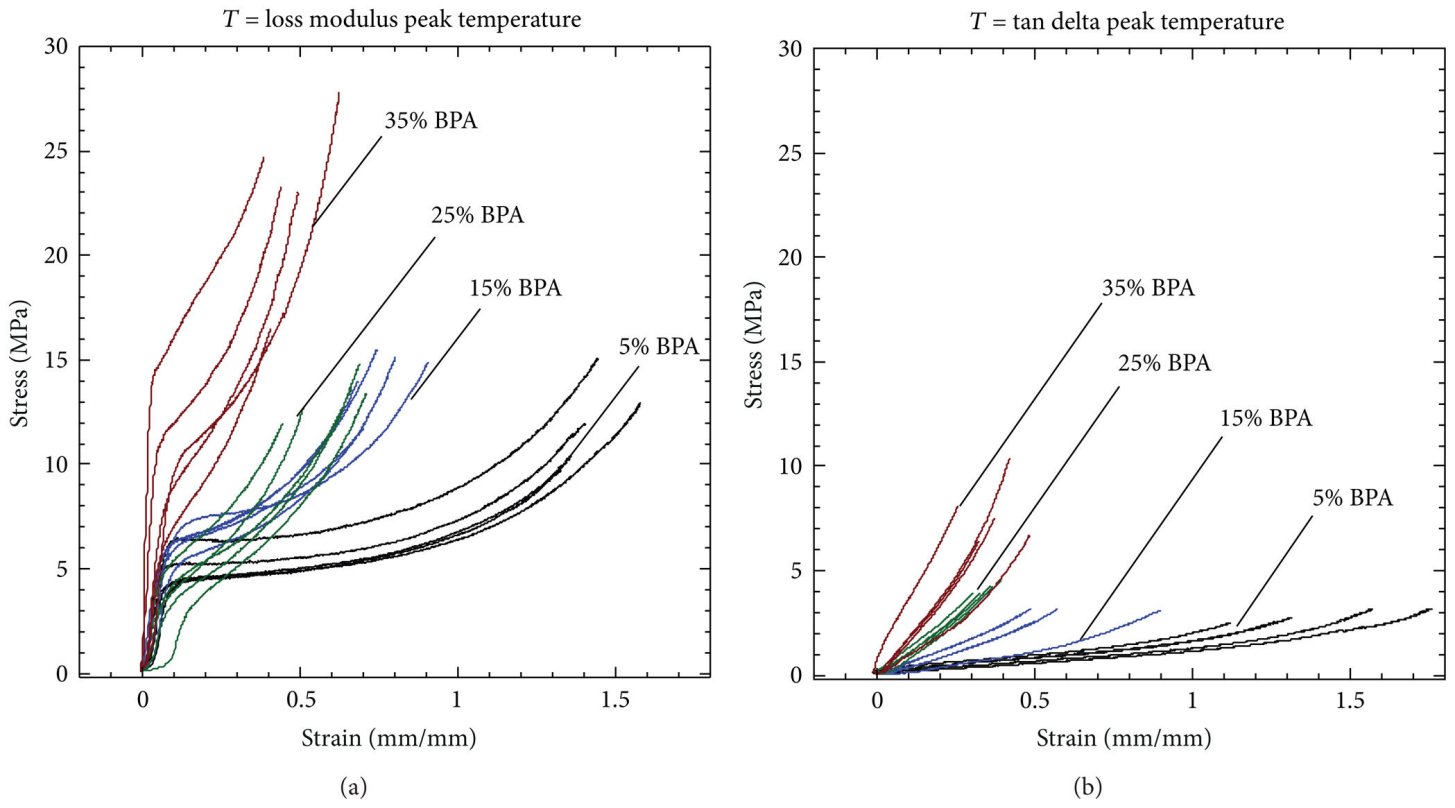
Tensile properties of the four copolymers measured at each copolymer’s loss modulus peak (a) and tan delta peak (b) temperatures. In both temperatures, the strain capacity of the copolymers decreased as a result of increasing crosslinker; however, a more brittle response was observed at tan delta peak temperatures.

Strain-to-failure experiments were performed on all four BPA composition samples at both loss modulus peak and tan delta peak temperatures. All stress/strain results are shown in Figures 7(a) and 7(b), and average stress-at-failure, strain-to-failure, and toughness (area under the stress/strain curves) data is listed in Table 2. For strain-to-failure experiments conducted at loss modulus peak temperatures, increasing the BPA crosslinker from 5 to 35 mole% resulted in an average stress-at-failure increase from 11.9 to 23.0 MPa and an average strain-to-failure decrease from 1.43 to 0.48 mm/mm. For strain-to-failure experiments conducted at tan delta peak temperatures, increasing BPA composition resulted in an average stress-at failure increase from 2.8 to 7.7 MPa and an average strain-to-failure decrease from 1.41 to 0.37 mm/mm. For both the loss modulus peak temperature and tan delta peak temperature strain-to-failure series, toughness decreased as BPA composition was increased from 5 to 15 to 25 mole% and then increased for the 35% BPA samples. One possible explanation for this increase in toughness for the most highly crosslinked samples is that bisphenol A groups have been shown to be capable of undergoing *π*-*π* stacking, which increases toughness in materials such as poly(bisphenol A carbonate) [32]. It is possible that, at high enough BPA compositions, *π*-*π* stacking can become predominant enough to improve toughness.

### 3.3. Device Crimping

**Figure 8:**
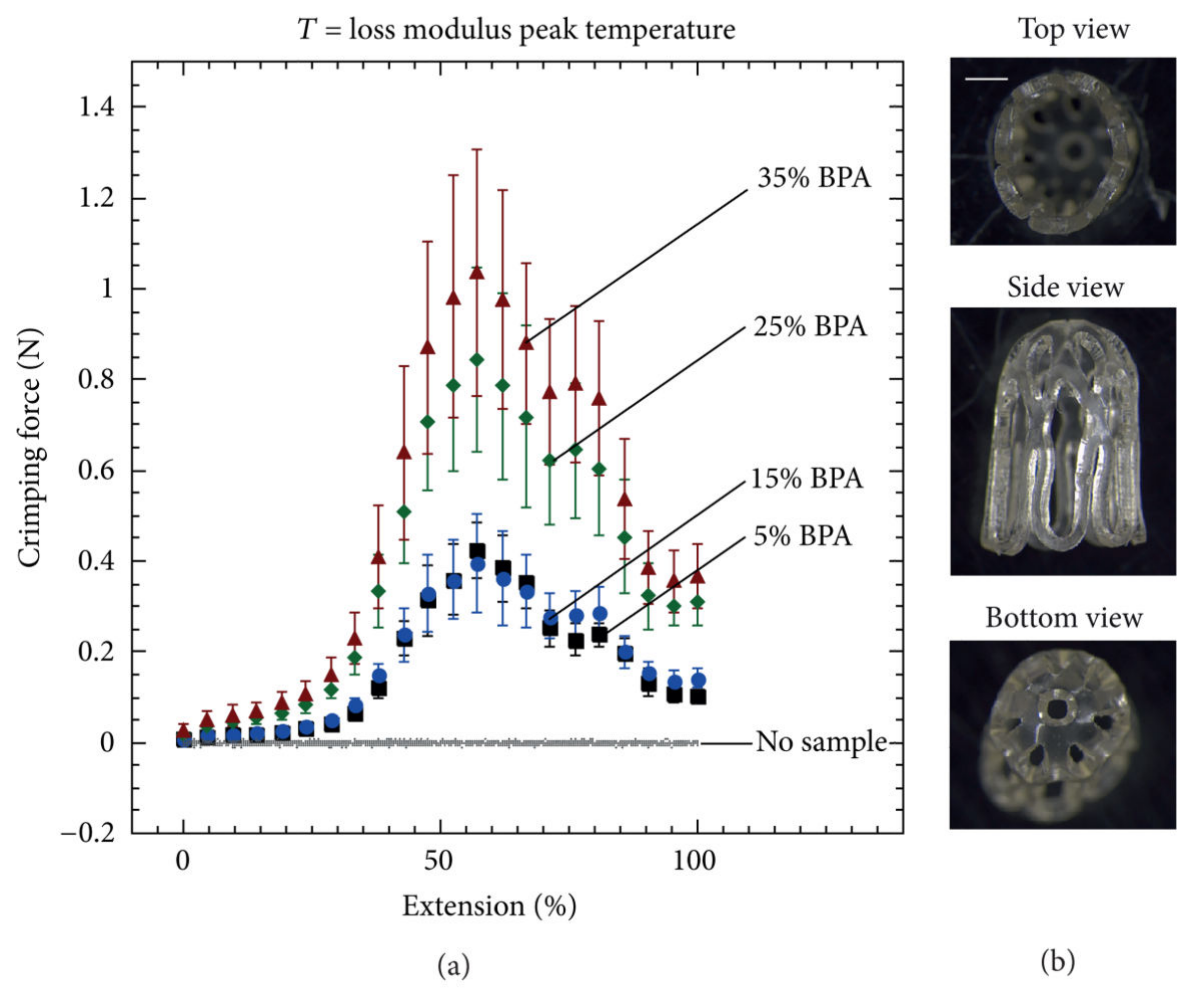
Force measurements of devices crimped at loss modulus peak temperature (a). Example of one device with 35 mole% BPA crimped at loss modulus peak temperature (scale bar = 1 mm) (b). Images were taken at 2.5x magnification.

**Figure 9:**
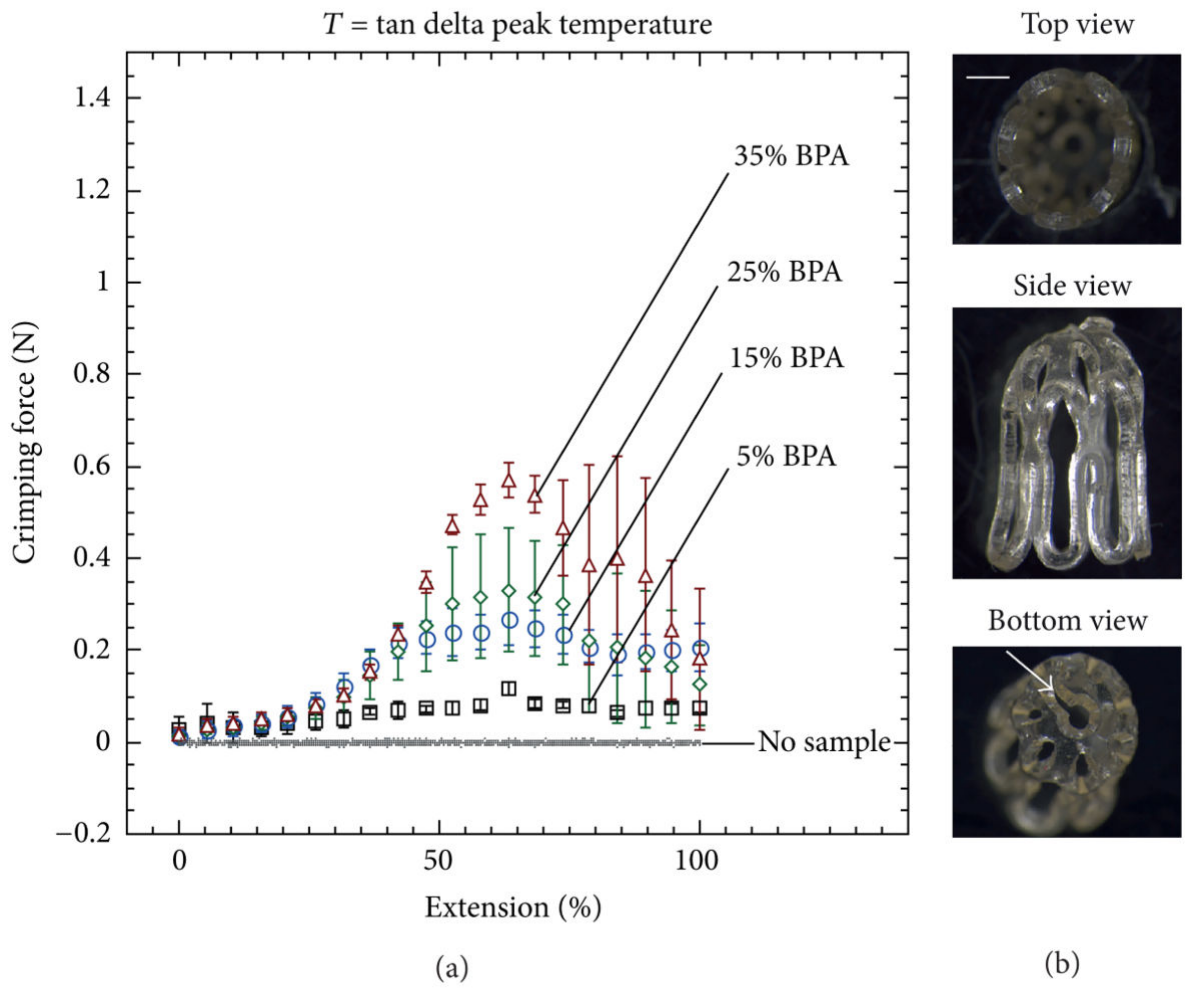
Force measurements of devices crimped at tan delta peak temperature (a). Example of one device with 35 mole% BPA crimped at tan delta peak temperature (scale bar = 1 mm) (b). Images were taken at 2.5x magnification. White arrow points at a common site for failure.

A total of five devices were crimped for each of the four BPA compositions. Figures 8(a) and 9(a) show plots of force versus extension during crimping at loss modulus peak temperatures and tan delta peak temperatures, respectively. Figures 8(b) and 9(b) show images of the top, side, and bottom views of crimped 35 mole% BPA devices at the two respective crimping temperatures. The crimping temperature had a significant effect on both the magnitude of the force required for crimping and on the propensity for device failure during crimping. For devices crimped at loss modulus peak temperature, the maximum average crimping force increased from 0.41 to 1.05N as BPA composition increased from 5 to 35 mole%. For devices crimped at tan delta peak temperature, the maximum average crimping force increased from 0.10 to 0.58N as BPA composition increased from 5 to 35 mole%. None of the devices crimped at loss modulus peak temperature failed upon crimping. On the contrary, the devices crimped at tan delta peak temperature were much more susceptible to failure during crimping: for the 5, 15, 25, and 35 mole% BPA samples, the number of devices that failed was 1, 2, 3, and 5, respectively. Most failure locations consistently occurred at the bases of the devices and appeared to initiate at the edges of the center holes, as shown in Figure 9(b).

### 3.4. Device Constrained Recovery

**Figure 10:**
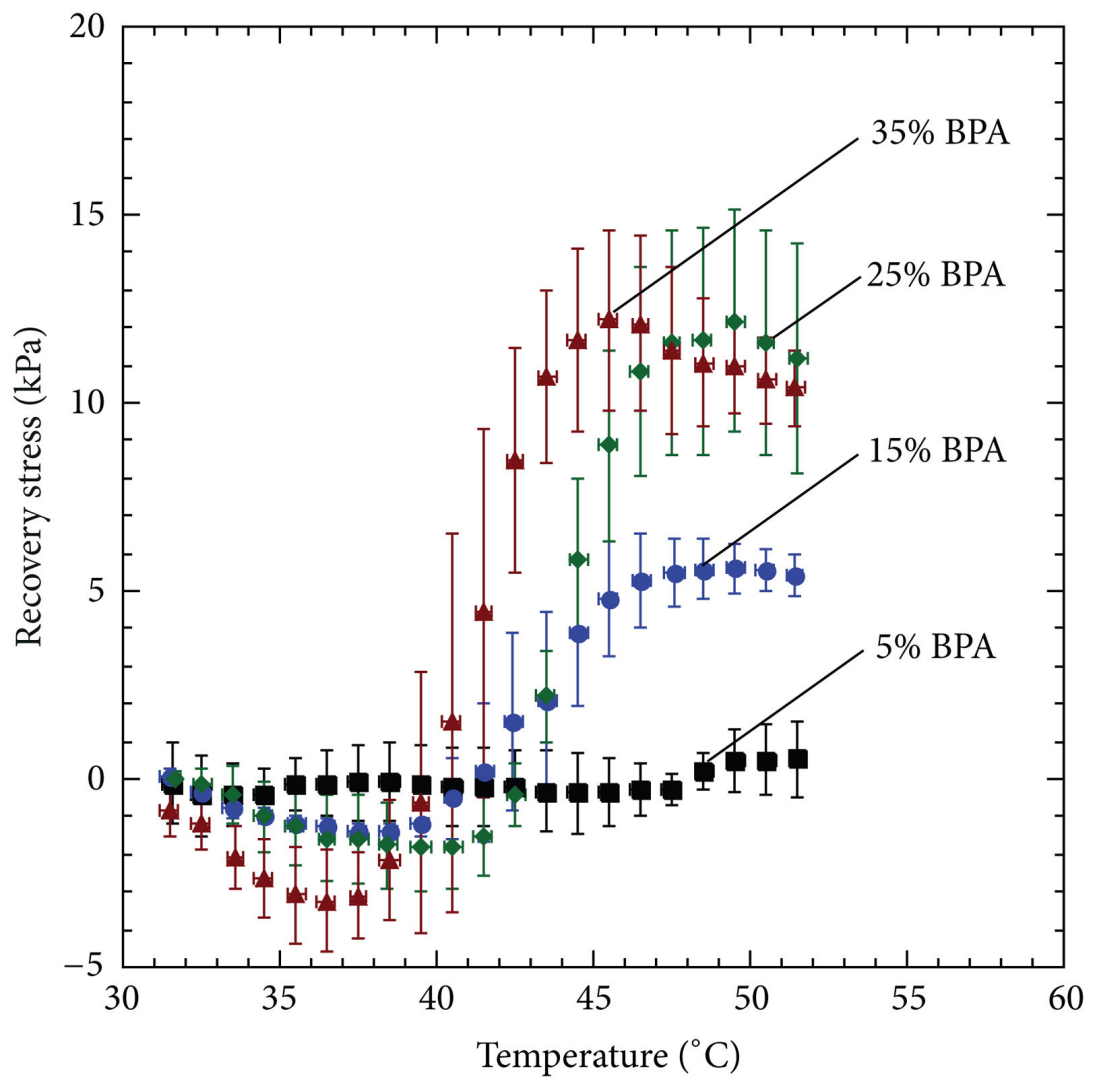
Recovery stress of thrombectomy devices during constrained recovery. Increasing the crosslinker amount resulted in an increase in recovery stress measured under constrained conditions. However, for the two highest crosslinked compositions, the recovery stress was not significantly different, because failures were observed in both compositions but were more prevalent in devices with 35 mole% BPA.

**Figure 11:**
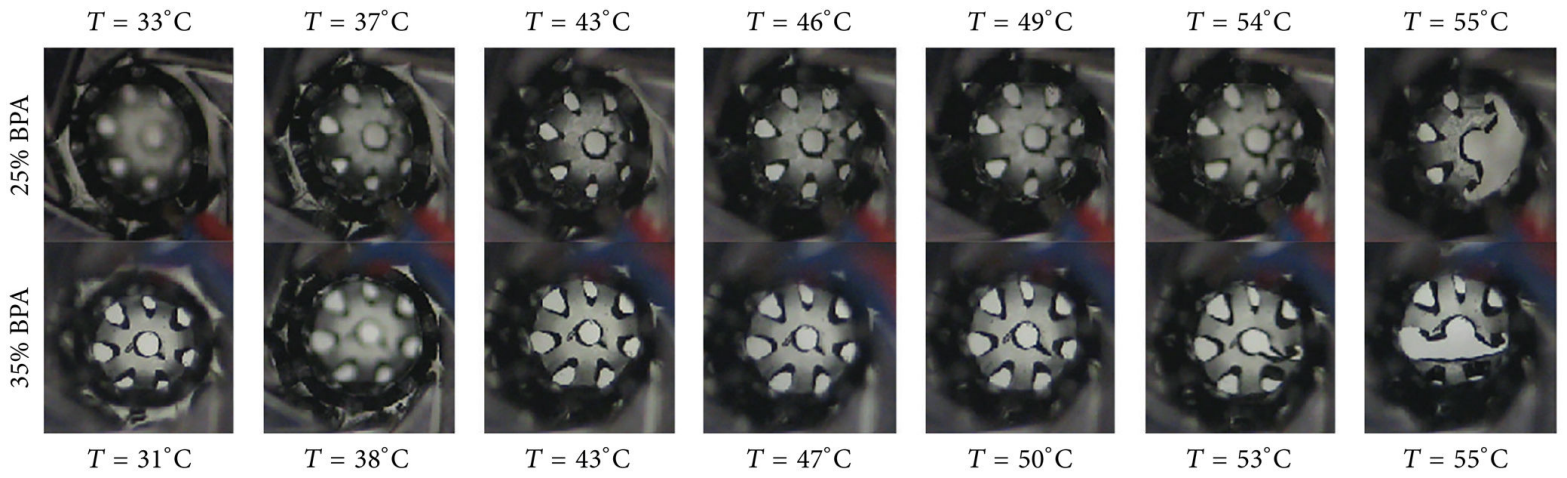
Crack development in devices of each composition (i.e., 25 and 35 mole% BPA) during constrained recovery test at various temperature points as the device was recovering its primary shape.

Because of the significant device failures that occurred during crimping at tan delta peak temperatures, only devices crimped at loss modulus peak temperatures were subjected to constrained recovery tests. Five devices were tested for each BPA composition. In Figure 10, a plot of average recovery stress versus temperature is shown for devices with varying BPA composition. As BPA composition was increased from 5 to 35 mole%, average recovery stress increased from 0.526 to 12.2 kPa. It is notable that during the constrained recovery tests, two out of the five 25 mole% BPA devices and four out of the five 35 mole% BPA devices developed cracks and subsequently broke. Images of crack development at different temperature points during constrained recovery of 25 and 35 mole% BPA samples are shown in Figure 11.

### 3.5. Blood Clot Removal Experiments

**Figure 12:**
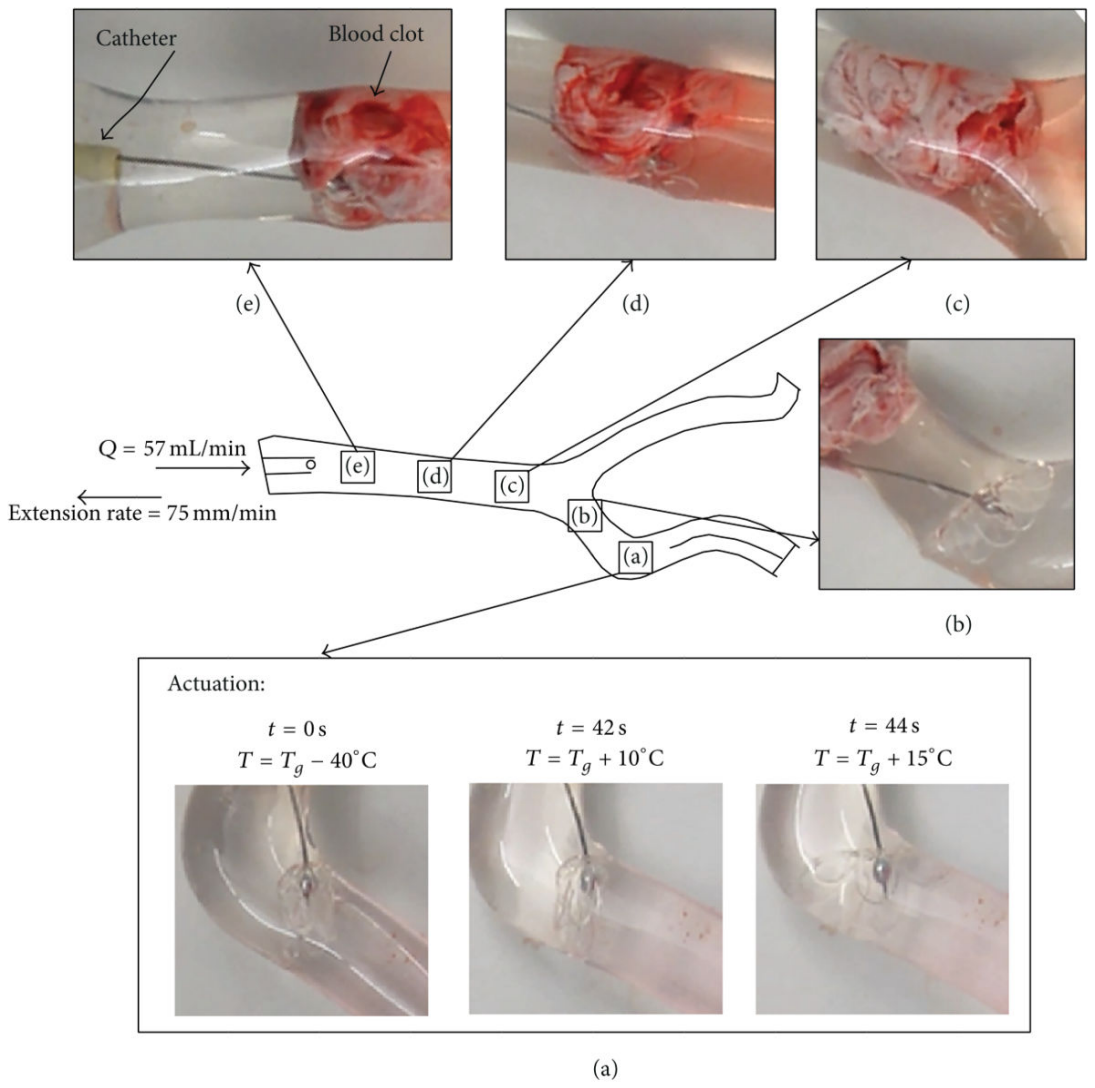
Actuation and clot removal with 15 mole% BPA device. *Q*: experimental flow rate (=57 mL/min). Panel (a) shows the device during actuation as the hot water was injected. Panels (b)–(e) depict the actuated thrombectomy device as it is withdrawn by the tensile tester successfully moving the blood clot from the occluded vessel (panel (b)) to the location of the catheter (panel (e)).

Three 5 and 15 mole% BPA devices were subjected to blood clot removal experiments with flow. In order to better simulate *in vivo* conditions, an experimental flow rate of 57 mL/min was chosen. This flow rate is equivalent to 113 mL/min after dynamic similarity via matching the Reynolds number, which is within the range of flow rates recorded in the middle cerebral artery, a common site for thromboembolic occlusion in the neurovasculature [31]. Because the 25 and 35 mole% BPA devices developed cracks during recovery under constrained conditions, they were not considered for the blood clot removal experiments. Images of the thrombectomy experiments for a 15 mole% BPA device and a diagram of the silicone vascular model used in the experiments are provided in Figure 12. To demonstrate that the crimped devices could maintain their crimped geometries under physiological conditions, they were first immersed in 37°C water and positioned past the bovine blood clot in the silicone vessel model, as pictured in Figure 12(a). After holding at 37°C to demonstrate shape fixity for 4–5 min, the water temperature was elevated to ~*T_g_* + 15 °C (maximum recorded temperature: 77°C) over the course of 45 s by injecting water near boiling temperature to the flow system. The benefit of increasing crosslink density on device blood clot removal was demonstrated: 0/3 of the 5 mole% BPA devices resulted in a successful extraction, while 2/3 of the 15 mole% BPA devices resulted in a successful blood clot extraction (i.e., the devices were able to move the blood clot from its origin at the bifurcation pictured in Figure 12(b) to the catheter tip at the end of the vasculature model, pictured in Figure 12(e)).

## 4. Discussion

The motivation for this study is the need for a SMP-based thrombectomy device that can exhibit sufficient recovery stress to reduce the likelihood of blood clot dislodgement during thrombectomy, while also being able to undergo sufficient deformation for catheter delivery. Thrombectomy is a promising treatment technique for the ~97% of ischemic stroke patients who are not eligible to receive tPA, the only FDA-approved drug for the treatment of ischemic stroke [2, 6].

Given the geometry of the device, recovery stress plays an important role in achieving blood clot entrapment and successful removal. The devices tested in the bench-top thrombotic stroke model in this study consisted of 5 and 15 mole% BPA. The average recovery stress of the 15 mole% BPA devices was roughly one order of magnitude higher than that of the 5 mole% BPA devices. This positive correlation between crosslink density and recovery stress in SMPs has been reported in numerous previous studies [11, 26, 27]. In an ideal rubber, elastic modulus is inversely proportional to the average molecular weight between crosslinks, so a more highly crosslinked rubber will have a higher stiffness than a more lightly crosslinked rubber. Consequently, more energy is required to achieve a specific deformation in amore highly crosslinked rubber than in a more lightly crosslinked rubber. Since the energy required to achieve these deformations is metastably stored when thermally actuated SMPs are strained and then cooled below their thermal transition regions, the force exerted by an SMP during recovery is consequently higher for a more highly crosslinked SMP [11, 25–27, 30]. None of the 5 mole% BPA devices (0/3) exerted enough recovery stress to hold the blood clot in our bench-top thrombotic stroke model, while 2/3 of the 15 mole% BPA devices did retain the blood clot as it was being removed.

The goal of varying crosslink density in this study was to increase device recovery stress for improved blood clot hold. However, increased crosslink density also had a negative impact on device mechanical integrity: the majority of the 25 and 35 mole% BPA samples did not endure the constrained recovery experiments. The temperature at which the devices were crimped also played an important role in device integrity. The strain-to-failure data in Figure 3 shows that the stress required to deform the specimens to specific strains decreased with increasing temperature for all compositions. Thus, it was expected that the forces required for crimping at higher temperatures (tan delta peak temperatures)were lower than those required for crimping at lower temperatures (loss modulus peak temperatures). Also, significant failure rates occurred during crimping at tan delta peak temperatures, while no failures occurred during crimping at loss modulus peak temperatures. This trend in failure during crimping most likely occurred because the samples were in higher toughness states at loss modulus peak temperatures than at tan delta peak temperatures (see Table 2). The devices that were more prone to failure at tan delta peak temperatures were the ones that exhibited strain-to-failure values below 0.4 mm/mm, and all failures originated at the base of the devices. This failure location is in accordance with FEA simulation predictions, which indicate that the bases of the devices are subjected to high localized strains within the range of 0.35 and 0.4 mm/mm. These localized strains, in conjunction with material limitations and possible machining defects, most likely caused crack initiations, which then led to device failures.

For the devices that were successfully crimped, the recovery stress under constrained conditions increased with increasing crosslink density. Nonetheless, some of the devices with 25 and 35 mole% BPA, which had rubbery moduli greater than 10MPa, failed during constrained recovery. In a similar manner to the findings in this study, Safranski and Gall [24] reported that for acrylic SMPs with rubbery moduli greater than 10MPa the crosslinking dominates the large strain mechanical properties of the network, and a relatively brittle response is observed. This pronounced brittle response in addition to machining defects may be the reason for failures in the devices with higher crosslink densities during constrained recovery.

Full constrained recovery experimentation represents a worst-case condition, but it is of great importance for a device such as the SMP thrombectomy device in this study. The pressure-strain elastic modulus of the common carotid artery, for example, is reported to be on the order of 55 kPa for healthy patients [33]. However, as patients’ age and arteries become more diseased, this value has been shown to increase to ~165 kPa [34]. Simply testing an actuating thrombectomy device in a silicone vessel model alone and neglecting to perform constrained recovery experiments could result in device failures such as the ones observed during the constrained recovery experiments in this study to remain unnoticed because a silicone vessel model is generally more compliant than a diseased artery. Thus, constrained recovery analysis should be performed a priori to avoid fatal complications that would probably only come into play in a more realistic environment, such as a diseased artery in a stroke patient.

## 5. Conclusions

We have reported an acrylic SMP system with glass transitions above body temperature in the range of 65 to 75°C with tailorable recovery stresses that were controlled by varying crosslink density. As expected, the increases in recovery stress that came with increasing crosslink density also resulted in reduced strain capacity and increased brittleness, which compromised the mechanical integrity of the devices during crimping and actuation. Thus, our results demonstrate that SMP materials with the highest rubbery moduli are not necessarily the best for applications requiring large deformations. From the four different material compositions evaluated, devices with 15 mole% BPA gave the most favorable outcome. However, only 2/3 of the devices of this composition were able to retain the blood clot in the bench-top experiment, suggesting that the system of acrylic SMPs presented in this study is not suitable for this application.

## Figures and Tables

**Table 1:** Rubbery modulus and mechanical transition temperature values.

Composition	Rubbery modulus (MPa)	Loss modulus peak temperature (°C)	Tan delta peak temperature (°C)
5% BPA	1.67 ± 0.18	62.3 ± 0.16	72.3 ± 0.16
15% BPA	5.92 ± 0.08	53.6 ± 0.17	66.9 ± 0.16
25% BPA	11.0 ± 0.32	50.8 ± 0.25	62.8 ± 0.24
35% BPA	15.6 ± 1.02	50.8 ± 0.11	61.5 ± 0.12

**Table 2:** Rubbery modulus and mechanical transition temperature values.

	Composition	Strain-to-failure (mm/mm)	Stress-at-failure (MPa)	Toughness (MJ/m^3^)
*T* = temperature at loss modulus peak	5% BPA	1.43 ± 0.0985	11.9 ± 2.14	8.99 ± 1.72
	15% BPA	0.773 ± 0.0906	14.2 ± 1.51	6.21 ± 0.992
	25% BPA	0.609 ± 0.119	13.2 ± 1.07	3.96 ± 0.747
	35% BPA	0.475 ± 0.0957	23.0 ± 4.15	6.38 ± 1.79

*T* = temperature at tan delta peak	5% BPA	1.41 ± 0.254	2.75 ± 0.338	1.49 ± 0.296
	15% BPA	0.590 ± 0.182	2.87 ± 0.254	0.697 ± 0.169
	25% BPA	0.357 ± 0.0354	4.09 ± 0.251	0.647±0.0723
	35% BPA	0.376 ± 0.0879	7.73 ± 1.56	1.24 ± 0.316
